# Draft Genome Sequence of *Leptospira interrogans* Serovar Bataviae Strain LepIMR 22 Isolated from a Rodent in Johor, Malaysia

**DOI:** 10.1128/genomeA.00956-16

**Published:** 2016-09-08

**Authors:** Fairuz Amran, Mohd Khairul Nizam Mohd Khalid, Saharuddin Mohamad, Adiratna Mat Ripen, Norazah Ahmad, Marga G. A. Goris, Ayu Haslin Muhammad, Nurul Atiqah Noor Halim

**Affiliations:** a Infectious Disease Research Centre, Bacteriology Unit, Institute for Medical Research, Kuala Lumpur, Malaysia; b Specialised Diagnostics Centre, Molecular Diagnostics and Protein Unit, Institute for Medical Research, Kuala Lumpur, Malaysia; c Institute of Biological Sciences, Faculty of Science, University of Malaya, Kuala Lumpur, Malaysia; d Primary Immunodeficiency Unit, Allergy and Immunology Research Centre, Institute for Medical Research, Kuala Lumpur, Malaysia; e KIT Biomedical Research, Royal Tropical Institute (KIT), Amsterdam, The Netherlands

## Abstract

*Leptospira interrogans* serovar Bataviae was recently identified as one of the persistent *Leptospira* serovars in Malaysia. Here, we report the draft genome sequence of the *L. interrogans* serovar Bataviae strain LepIMR 22 isolated from kidney of a rodent in Johor, Malaysia.

## GENOME ANNOUNCEMENT

Leptospirosis is a zoonotic infection with higher incidence in tropical regions and has been recognized as one of the emerging infectious diseases worldwide (1). The genus *Leptospira* contains at least 21 species, with the pathogenic *Leptospira* spp. being further classified into over 250 different serotypes (2). Recently, *Leptospira borgpetersenii* serovar Javanica and *Leptospira interrogans* serovar Bataviae were identified as the persistent serovars in the urban rat populations in Malaysia (3). Both serovars were further genotyped by multilocus sequence typing (MLST) into sequence type 143 (ST143) for Javanica and ST50 for Bataviae (4). The availability of genome sequences from both *Leptospira* serovars could therefore provide very useful information, especially in identifying potential targets for leptospiral vaccines in Malaysia.

Here, we present the draft genome of *L. interrogans* serovar Bataviae strain LepIMR 22, isolated from kidney of a rodent in Johor, Malaysia. The bacteria was cultured into EMJH medium with daily inspection under a dark-field microscope. Both 16S rRNA and serotyping were used to characterize the isolate as *L. interrogans* serovar Bataviae, with the serotyping performed in the Royal Tropical Institute (KIT), Amsterdam, The Netherlands. Genomic DNA was extracted using the QIAamp DNA minikit, according to the manufacturer’s protocol. Sequencing was performed using the Illumina MiSeq system with 2 × 250-bp paired-end chemistry.

Sequencing resulted in 5,811,520 reads, with 4,400,961 reads passing the sequence trimming (*Q* ≥ 30) and filtering (length, ≥25 bp). *De novo* assembly was performed using CLC Genomics Workbench 6.0.1 and resulted in 332 contigs of approximately 97-fold coverage, with an *N*_50_ of 34,001. The LepIMR 22 draft genome comprises ~4.72 Mbp, with an overall G+C content of 35.19%. Gene prediction was performed using Prodigal version 2.60 (5), while tRNAs and rRNAs were predicted using tRNAscan-SE version 1.3.1 (6) and RNAmmer version 1.2 (7), respectively. Open reading frames (ORFs) found by Prodigal were subjected to a similarity search using BLAST version 2.2.25+ against the NCBI GenBank nonredundant protein sequence database. A multilocus sequence typing (MLST) analysis was performed using a publicly available MLST server (https://cge.cbs.dtu.dk//services/MLST/) that uses whole-genome sequencing (WGS) data to identify the STs of bacteria (8).

In total, 3,917 coding sequences, 37 tRNAs, and three rRNAs were identified. MLST analysis identified the LepIMR 22 strain as ST50.

### Accession number(s).

This whole-genome shotgun project has been deposited in DDBJ/ENA/GenBank under the accession no. LUVO00000000.
